# The homeostasis model assessment of insulin resistance is a judgment criterion for metformin pre-treatment before IVF/ICSI and embryo transfer cycles in patients with polycystic ovarian syndrome

**DOI:** 10.3389/fendo.2023.1106276

**Published:** 2023-02-09

**Authors:** Rui Gao, Lang Qin, Zhengyu Li, Wenjiao Min

**Affiliations:** ^1^ The Reproductive Medical Center, Department of Gynecology and Obstetrics, West China Second University Hospital, Sichuan University, Chengdu, China; ^2^ Key Laboratory of Birth Defects and Related Diseases of Women and Children, Sichuan University, Ministry of Education, Chengdu, China; ^3^ Department of Gynecology and Obstetrics, West China Second University Hospital, Sichuan University, Chengdu, China; ^4^ Psychosomatic Department, Sichuan Provincial People’s Hospital, University of Electronic Science and Technology of China, Chinese Academy of Sciences Sichuan Translational Medicine Research Hospital, Chengdu, China

**Keywords:** polycystic ovary syndrome, *in vitro* fertilization, intracellular sperm injection, embryo transfer, metformin, insulin resistance, HOMA-IR, clinical pregnancy rate

## Abstract

**Purpose:**

The aim of this study was to explore the value of the homeostasis model assessment of IR (HOMA-IR) as a judgment criterion for metformin pre-treatment before *in vitro* fertilization/intracellular sperm injection (IVF/ICSI) and embryo transfer (ET) for polycystic ovarian syndrome (PCOS) patients.

**Materials and methods:**

The clinical and laboratory information of PCOS patients who received IVF/ICSI-ET from January 2017 to September 2021 was retrospectively analyzed. We compared the clinical pregnancy rate (primary outcome) and controlled ovarian stimulation (COS)-related parameters (secondary outcomes) between patients with and without metformin pre-treatment for all PCOS patients not grouped by HOMA-IR, PCOS patients with HOMA-IR < 2.71, and PCOS patients with HOMA-IR ≥ 2.71.

**Results:**

A total of 969 PCOS patients who received the GnRH-antagonist protocol were included in this study. For all PCOS patients, the metformin group showed comparable clinical pregnancy rates in fresh ET cycles and frozen ET cycles compared with the control group (55.9% vs. 57.1%, *p* = 0.821 and 63.8% vs. 60.9%, *p* = 0.497). For PCOS patients with HOMA-IR < 2.71, the clinical pregnancy rates in both fresh ET cycles and frozen ET cycles were statistically similar between the two groups (61.5% vs. 57.6%, *p* = 0.658 and 70.6% vs. 66.7%, *p* = 0.535). For PCOS patients with HOMA-IR ≥ 2.71, the clinical pregnancy rate in fresh ET cycles was comparable between the two groups (51.5% vs. 56.3, *p* = 0.590), but it was statistically higher in the metformin group than in the control group in frozen ET cycles (57.1% vs. 40.0%, *p* = 0.023). The metformin group had less oocytes retrieved, a lower cleaved oocyte rate, a lower available D3 embryo rate, a lower blastocyst formation rate, and a lower available blastocyst rate than the control group.

**Conclusion:**

HOMA-IR is a judgment criterion for metformin pre-treatment before IVF/ICSI-ET in patients with PCOS. Metformin pre-treatment could be added for PCOS patients with HOMA-IR ≥ 2.71 during frozen IVF/ICSI-ET cycles to improve the clinical pregnancy rate.

## 1 Introduction

Polycystic ovary syndrome (PCOS) is an endocrinological problem that affects about 5%–10% of women of childbearing age all over the world (1). Oligo-anovulation, clinical and/or biochemical hyperandrogenism, and polycystic ovary morphology (PCOM) on ultrasonography are the most important clinical manifestations of PCOS (2). It was reported that approximately 80% of anovulation infertility was caused by PCOS (3). *In vitro* fertilization/intracellular sperm injection (IVF/ICSI)–embryo transfer (ET) is a third-line method that helps PCOS patients who are unable to conceive after adjusting lifestyle and medication treatment (3). However, performing IVF/ICSI-ET for PCOS patients is full of challenge because of the poorer-quality embryos, the higher risk of ovarian hyper-stimulation syndrome (OHSS), and the lower clinical pregnancy rate compared with healthy women (4).

IR is defined as reduced insulin sensitivity, and an increased amount of insulin is needed to realize its normal function; the hyperinsulinemic–euglycemic clamp (HEC) technique was considered to be the gold standard for measuring insulin sensitivity (5). As previously reported, the incidence of IR among PCOS patients varies from 50% to 70% in different regions (6). IR was reported as an important risk factor of failure of IVF/ICSI-ET for PCOS patients, because it may impair the oocyte development/maturation and reduce endometrial receptivity (7). The homeostasis model assessment of IR (HOMA-IR) is widely used to assess the degree of IR (8). Metformin is a common insulin-sensitizing agent, but the application of metformin on PCOS patients undergoing IVF/ICSI-ET is full of controversy. A systematic review and meta-analysis showed that metformin may reduce live birth rate during the GnRH-antagonist protocol, but its effect on the clinical pregnancy rate was uncertain (9). At present, the studies focusing on the association between metformin pre-treatment and IVF/ICSI-ET outcomes were heterogeneous. In particular, there are a few studies that are focused on the influence of HOMA-IR on outcomes of metformin pre-treatment in PCOS patients undergoing IVF/ICSI-ET.

We speculated that a different HOMA-IR reflects the different efficacies of metformin pre-treatment on IVF/ICSI-ET among PCOS patients. To confirm this conjecture, we performed this retrospective study to explore the value of HOMA-IR as a judgment criterion for metformin pre-treatment before IVF/ICSI-ET cycles in patients with PCOS.

## 2 Materials and methods

### 2.1 Participants and study design

A single-center retrospective cohort study was performed at West China Second University Hospital, Sichuan University from January 2017 to September 2021. The study was approved by the Ethics Committee of West China Second University Hospital. PCOS patients who received IVF/ICSI-ET because of anovulation infertility or male infertility were included in this study. Sociodemographic information, clinical manifestations, laboratory indicators, and treatment information of these PCOS patients were collected from the electronic medical record management system. The diagnosis of PCOS was based on the Rotterdam criteria, which required that at least two of the following three criteria were satisfied: oligomenorrhea or anovulation, clinical or biochemical hyperandrogenism, and polycystic ovaries on ultrasonography (defined as either an ovary with antral follicle count ≥ 12 or an ovarian volume ≥ 10 cm^3^); other causes of hyperandrogenism and ovulation dysfunction were excluded (2).

The exclusion criteria were as follows: (1) incomplete sociodemographic information, clinical manifestations, laboratory indicators, or treatment information; (2) patients with other factors of infertility such as Asherman’s syndrome, submucosal fibroids of the uterus, and other uterine malformations; (3) patients with other endocrine diseases such as thyroid diseases, diabetes mellitus, and hyperprolactinemia; and (4) patients with a history of recurrent spontaneous abortion. All included patients were checked again for their metformin pre-treatment protocols. Patients who received a metformin pre-treatment form at least 3 months before their IVF/ICSI-ET cycles constituted the metformin group; patients without metformin pre-treatment made up the control group, and other patients were excluded. The subgroup analyses were based on HOMA-IR < 2.71 and HOMA-IR ≥ 2.71 according to a previous study, which reported that HOMA-IR ≥ 2.71 is a significant risk factor of adverse pregnancy outcomes for women who received fresh IVF/ICSI-ET (10).

### 2.2 Controlled ovarian stimulation and embryo transfer

All included PCOS patients underwent a GnRH-antagonist protocol for controlled ovarian stimulation (COS); they were started on intramuscular injections of recombinant FSH (Injection Gonal-F, Merck Serono Specialties, Italy) or human menopausal gonadotropin (hMG, Lizhu Pharmaceutical Trading, China) from the second day of their menstrual cycle. The starting dose was between 150 IU/day and 225 IU/day. A GnRH antagonist (Injection Cetrotide acetate, Aeterna Zentaris, Canada) was administered at a dose of 0.25 mg/day from the sixth day of the menstrual cycle until the ovulation trigger day. The cycles were cancelled in patients with no follicle greater than 10 mm after 10 days of recombinant FSH/hMG stimulation. For all PCOS patients, when at least two follicles are ≥18 mm or three follicles ≥17 mm, the final stage of triggering ovulation was performed using human chorionic gonadotropin (hCG; Lizhu Pharmaceutical Trading, China) at doses from 8,000 IU to 10,000 IU. For patients at a high risk for ovarian hyper-stimulation syndrome (OHSS), 4,000 IU to 5,000 IU of hCG was used to trigger ovulation.

Oocyte retrieval was performed 36–38 h after triggering ovulation. Oocyte assessment was performed by the standard morphology criteria, and nuclear maturity assessment was performed. Conventional IVF or ICSI was performed depending on semen parameters. For patients who received fresh ET cycles, ET was performed 3 or 5 days after oocyte retrieval according to the type of embryo; other embryos were all frozen. For patients who received their first frozen ET cycles, artificial menstrual cycle was established by the exogenous addition of estradiol and ET was performed 3 or 5 days after the addition of progesterone. All patients were given luteal phase support *via* the intramuscular injection of progesterone (100 mg/day) or vaginal progesterone gel (90 mg/day) plus oral dydrogesterone (20 mg/day). Two weeks after ET, pregnancy was assessed by serum β-hCG levels and confirmed by transvaginal ultrasound 4 weeks after ET. Serum β-hCG levels > 50 IU/L were regarded as biochemical pregnancy and the presence of the gestational sac was regarded as clinical pregnancy.

### 2.3 Information collection and outcomes

Fasting plasma glucose (FPG) and fasting insulin (FINS) measured before metformin treatment were collected, and HOMA-IR was calculated with the following formula: HOMA-IR = FGP (mmol/L) FINS (mIU/L)/22.5. We summed up the basic information of patients, including their fertility history, clinical manifestations of PCOS, age, body mass index (BMI), and history of oral contraceptives (OC) treatment. BMI was calculated by the body weight in kilograms divided by the square of the height in meters (kg/m^2^). Venous blood samples were taken 2–4 days during a spontaneous menstrual cycle or an independent cycle phase in the presence of amenorrhea before metformin pre-treatment to detect anti-Müllerian hormone (AMH), dehydroepiandrosterone (DHEAS), androstenedione (ASD), follicle-stimulating hormone (FSH), luteinizing hormone (LH), estradiol (E2), progesterone (P), testosterone (T), and sex hormone binding globulin (SHBG) levels. At the same time, homocysteine (HCY), high-density lipoprotein cholesterol (HDL-C), and triglycerides (TG) were also assessed after an overnight fast of at least 10 h.

COS-related parameters were also collected, including OHSS rate, the type of gonadotropin (Gn), the starting dosage of Gn, the total number of Gn days, and the total Gn dosage. Serum LH, E2, and P levels were detected and single endometrial thickness (ET) was measured by ultrasonography on the trigger day. The number of follicles ≥14 mm on the trigger day and the number of oocytes retrieved were also collected. Embryo grading was done by a standard morphology assessment according to modified Veeck’s scoring. The IVF/ICSI fertilization rate, cleavage rate, available D3 embryo rate, high-quality D3 embryo rate, blastocyst formation rate, available blastocyst rate, and high-quality blastocyst rate were calculated and were selected as secondary outcomes of this study. Clinical pregnancy rate was defined as the presence of a gestational sac per ET cycle and was selected as the primary outcome of this study.

### 2.4 Statistical analysis

We used a Kolmogorov–Smirnov test to estimate whether data were normally distributed. Normally distributed continuous variables were presented as means ± standard deviations (SDs) and were analyzed by *t*-test. Non-normally distributed continuous variables were presented as median (25th–75th percentiles) and were analyzed by the Kruskal–Wallis test. Categorical measurements were presented as a percentage and were compared by chi-squared test; if numbers were less than 5 in at least 20% of the cells, Fisher’s exact test was performed. The adjusted difference in the clinical pregnancy rate between the two groups was expressed as odds ratio (OR), 95% confidence intervals (CIs), and adjusted *p*-value. *p*-value < 0.05 was regarded as statistically different. All the statistical analyses were performed by SPSS, version 26.0 (SPSS Inc., Chicago, IL, UPL).

## 3 Results

### 3.1 Basic information of PCOS patients

PCOS patients who entered IVF/ICSI-ET cycles because of anovulation infertility or male infertility were searched in the electronic medical record management system. After exclusion, 969 PCOS patients were included in this study. Among them, 366 patients were in the metformin group and 603 patients were in the control group. There was a statistical difference in BMI and history of OC treatment between the two groups (*p* < 0.05). For patients who received metformin pre-treatment, the HOMA-IR of 171 patients (46.7%) was <2.71 and that of 195 patients (53.3%) was ≥2.71. For patients in the control group, the HOMA-IR of 435 patients (72.1%) was <2.71 and that of 168 patients (27.9%) was ≥2.71.

Compared with the control group, the metformin group had lower levels of AMH, basal P, basal FSH, basal LH, and SHBG, and higher levels of HCY and TG. In addition, the metformin group had a higher total dosage of Gn, a shorter duration of Gn, and lower E2 levels on the trigger day than the control group. A detailed information of PCOS patients in the metformin group and control group is shown in **Table 1**. For PCOS patients with HOMA-IR < 2.71, the metformin group had a higher age, BMI, duration of infertility, FAI, HCY, and TG, and lower basal FSH levels than the control group. The proportion of history of OC treatment and type of Gn were significantly different between the two groups (as shown in **Supplementary Table 1**). For PCOS patients with HOMA-IR ≥ 2.71, the metformin group had lower basal FSH and basal LH levels, and higher basal T, FAI, and HDL-C levels than the control group. The proportion of PCOM and type of Gn were significantly different between the two groups (as shown in **Supplementary Table 2**).

**Table 1:** Baseline information and laboratory data between the metformin group and the control group.

	Metformin group (*n* = 366)	Control group (*n* = 603)	*p*-value
Age (year)	30 ± 4	29 ± 4	0.036
BMI (kg/m^2^)	23.60 ± 3.34	22.64 ± 3.41	<0.001
Duration of infertility (years)	4 ± 3	3 ± 2	0.001
HOMA-IR [*n* (%)]			<0.001
<2.71	171 (46.7)	435 (72.1)	
≥2.71	195 (53.3)	168 (27.9)	
Type of infertility [*n* (%)]			0.285
Primary	249 (68.0)	390 (64.7)	
Secondary	117 (32.0)	213 (35.3)	
PCOM [*n* (%)]	303/366 (82.8)	519/603 (86.1)	0.167
history of OC treatment [*n* (%)]	279/366 (76.2)	171/603 (28.4)	<0.001
AMH (ng/ml)	9.81 ± 4.77	10.46 ± 5.05	0.046
Basal E2 (pg/ml)	41.4 ± 15.5	42.8 ± 17.0	0.191
Basal P (ng/ml)	0.48 ± 0.19	0.52 ± 0.24	0.009
Basal FSH (IU/L)	6.2 ± 1.8	6.8 ± 1.8	<0.001
Basal LH (IU/L)	8.6 ± 5.8	9.5 ± 5.9	0.026
Basal T (mg/dl)	0.53 ± 0.26	0.46 ± 0.66	0.070
Basal DHEAS (μg/dl)	243.8 ± 117.1	236.5 ± 117.0	0.447
Basal ASD (ng/ml)	3.72 ± 1.36	3.78 ± 1.39	0.590
Basal SHBG (nmol/L)	33.9 ± 24.0	53.0 ± 36.8	<0.001
FAI	4.7 ± 3.9	4.7 ± 3.9	<0.001
HCY (μmol/L)	9.58 ± 2.35	9.18 ± 2.40	0.014
HDL-C (mmol/L)	1.34 ± 0.36	1.32 ± 0.33	0.450
TG (mmol/L)	2.06 ± 1.94	1.67 ± 1.77	0.002
Type of Gn [*n* (%)]			<0.001
rFSH	168 (45.9)	372 (61.7)	
hMG	198 (54.1)	231 (38.3)	
Starting dosage of Gn (IU)	178.0 ± 52.8	178.7 ± 55.6	0.848
Total dosage of Gn (IU)	1,933.2 ± 803.9	1,807.1 ± 711.2	0.011
Duration of Gn (days)	11 ± 2	10 ± 2	<0.001
On the trigger day			
E2 (pg/ml)	4,468.8 ± 2,688.7	5,166.2 ± 2,977.1	<0.001
P (ng/ml)	1.08 ± 0.92	1.15 ± 0.55	0.123
LH (IU/L)	2.7 ± 2.1	2.7 ± 2.1	0.949
Single endometrium thickness (mm)	5.1 ± 1.0	5.3 ± 1.1	0.059
No. of follicles ≥ 14 mm	10 ± 4	10 ± 3	0.137
Fertility methods [*n* (%)]			0.002
IVF	327 (89.3)	486 (80.6)	
ICSI	9 (2.5)	30 (5.0)	
IVF+ICSI	30 (8.2)	87 (14.4)	

BMI, body mass index; HOMA-IR, the homeostasis model assessment of insulin resistance; PCOM, polycystic ovarian morphology; OC, oral contraceptives; AMH, anti-Mullerian hormone; E2, estradiol; P, progesterone; FSH, follicle-stimulating hormone; LH, luteinizing hormone; T, testosterone; DHEAS, dehydroepiandrosterone sulfate; ASD, androstenedione; SHBG, sex hormone binding globulin; FAI, free androgen index; HCY, homocysteine; HDL-C, high-density lipoprotein cholesterol; TG, triglycerides; Gn, gonadotropin; rFSH, recombinant FSH; hMG, human menopausal gonadotropin; IVF, in vitro fertilization; ICSI, intracellular sperm injection. p < 0.05 was regarded as statistical different.

### 3.2 Metformin pre-treatment for all PCOS patients

We compared the COS-related parameters and clinical pregnancy rates between the metformin group and the control group for all PCOS patients. The results showed that the metformin group had fewer oocytes retrieved (15 ± 7 vs. 16 ± 8, *p* = 0.004), a lower cleaved oocyte rate (97.9% vs. 98.5, *p* = 0.013), a lower available D3 embryo rate (68.9% vs. 73.4%, *p* < 0.001), a lower blastocyst formation rate (69.3% vs. 71.7%, *p* = 0.043), and a lower available blastocyst rate (85.9% vs. 90.8%, *p* < 0.001) than the control group. However, there was no statistical difference in IVF fertilization rate (*p* = 0.726), ICSI fertilization rate (*p* = 0.294), high-quality D3 embryo rate (*p* = 0.092), and high-quality blastocyst rate (*p* = 0.749) between the two groups. We did not find a statistical difference in severe OHSS rate between the two groups (*p* = 0.372).

For 345 PCOS patients who received fresh ET cycles (177 patients in the metformin group and 168 patients in the control group), the clinical pregnancy rate between the metformin group and the control group was comparable (55.9% vs. 57.1%, *p* = 0.821). For 552 PCOS patients who received their first frozen ET cycles (207 patients in the metformin group and 345 patients in the control group), there was no statistical difference in the clinical pregnancy rate between the two groups (63.8% vs. 60.9%, *p* = 0.497). The above results are shown in **Table 2**. After adjusting for age, BMI, duration of infertility, history of OC treatment, AMH, basal FSH, basal LH, FAI, HCY, and TG, as these factors were statistically different between the two groups and were reported to be associated with the clinical pregnancy rate in IVF/ICSI-ET before, there was no statistical difference in the clinical pregnancy rate between the two groups either (as shown in **Supplementary Table 3**).

**Table 2:** COS outcomes and clinical pregnancy outcomes between the metformin group and the control group in all PCOS patients.

	Metformin group (*n* = 366)	Control group (*n* = 603)	*p*-value
No. of oocytes retrieved	15 ± 7	16 ± 8	0.004
IVF fertilization rate [*n* (%)]	4,137/4,395 (94.1)	6,639/7,065 (94.0)	0.726
ICSI fertilization rate [*n* (%)]	228/255 (89.4)	984/1131 (87.0)	0.294
Cleaved oocyte rate [*n* (%)]	4,275/4,365 (97.9)	7,512/7,623 (98.5)	0.013
Available D3 embryo rate [*n* (%)]	2,946/4,275 (68.9)	5,514/7,512 (73.4)	<0.001
High-quality D3 embryo rate [*n* (%)]	1,530/3,219 (47.5)	2,790/5,649 (49.4)	0.092
Blastocyst formation rate [*n* (%)]	1,386/2,001 (69.3)	3,093/4,311 (71.7)	0.043
Available blastocyst rate [*n* (%)]	1,191/1,386 (85.9)	2,808/3,092 (90.8)	<0.001
High-quality blastocyst rate [*n* (%)]	414/1,386 (29.9)	909/3,092 (29.4)	0.749
Severe OHSS rate [*n* (%)]	9/366 (2.5)	21/603 (3.5)	0.372
CPR in fresh cycle [*n* (%)]	99/177 (55.9)	96/168 (57.1)	0.821
CPR in frozen cycle [*n* (%)]	132/207 (63.8)	210/345 (60.9)	0.497

IVF, in vitro fertilization; ICSI, intracytoplasmic sperm injection; OHSS, ovarian hyper-stimulation syndrome; CPR, clinical pregnancy rate. p < 0.05 was regarded as statistical different.

### 3.3 Metformin pre-treatment for PCOS patients with HOMA-IR < 2.71

We did not find any statistical difference in IVF fertilization rate, ICSI fertilization rate, cleaved oocyte rate, high-quality D3 embryo rate, blastocyst formation rate, and high-quality blastocyst rate between the metformin group and the control group for PCOS patients with HOMA-IR < 2.71. However, there was a significantly lower available D3 embryo rate (66.1% vs. 72.3%, *p* < 0.001) and available blastocyst rate (84.8% vs. 91.3%, *p* < 0.001) in the metformin group compared with the control group. We found no statistical difference in severe OHSS rate between the two groups (*p* = 0.836).

The clinical pregnancy rate between the metformin group and the control group was comparable for 198 PCOS patients who received fresh ET cycles (61.5% vs. 57.6%, *p* = 0.658). There was no statistical difference in the clinical pregnancy rate between the metformin group and the control group (70.6% vs. 66.7%, *p* = 0.535) for 372 PCOS patients who received their first frozen ET cycles (102 patients in the metformin group and 270 patients in the control group). The above results are shown in **Table 3**. After adjusting for age, BMI, duration of infertility, history of OC treatment, basal FSH, FAI, HCY, and TG, there was no statistical difference in the clinical pregnancy rate between the two groups either (as shown in **Supplementary Table 4**).

**Table 3:** COS outcomes and clinical pregnancy outcomes between the metformin group and the control group in PCOS patients of different HOMA-IR subgroups.

	HOMA-IR < 2.71			HOMA-IR ≥ 2.71		
	Metformin group (*n* = 171)	Control group (*n* = 435)	*p*-value	Metformin group (*n* = 195)	Control group (*n* = 168)	*p*-value
No. of oocytes retrieved	15 ± 8	16 ± 8	0.300	14 ± 6	16 ± 8	0.004
IVF fertilization rate [*n* (%)]	1,995/2,135 (93.4)	4,581/4,866 (94.1)	0.259	2,142/2,256 (94.9)	2,058/2,199 (93.6)	0.051
ICSI fertilization rate [*n* (%)]	111/132 (84.1)	804/930 (86.5)	0.462	117/123 (92.7)	180/201 (89.6)	0.078
Cleaved oocyte rate [*n* (%)]	2,070/2,106 (98.3)	5,304/5,385 (98.5)	0.520	2,205/2,259 (97.5)	2,208/2,238 (98.7)	0.009
Available D3 embryo rate [*n* (%)]	1,368/2,070 (66.1)	3,834/5,304 (72.3)	<0.001	1,578/2,205 (71.6)	1,680/2,208 (76.1)	0.001
High-quality D3 embryo rate [*n* (%)]	729/1,539 (47.4)	1,971/4,044 (48.7)	0.360	801/1,680 (47.7)	819/1,605 (51.0)	0.055
Blastocyst formation rate [*n* (%)]	651/930 (70.0)	2,133/2,943 (72.5)	0.143	735/1,701 (43.2)	690/1,368 (50.4)	<0.001
Available blastocyst rate [*n* (%)]	552/651 (84.8)	1,947/2,133 (91.3)	<0.001	639/735 (86.9)	861/960 (89.7)	0.079
High-quality blastocyst rate [*n* (%)]	189/651 (29.0)	615/2,133 (28.8)	0.922	225/735 (30.6)	294/960 (30.6)	0.995
Severe OHSS rate [*n* (%)]	9/171 (5.3)	21/435 (4.8)	0.836	–	–	–
CPR in fresh cycle [*n* (%)]	48/78 (61.5)	69/120 (57.6)	0.658	51/99 (51.5)	27/48 (56.3)	0.590
CPR in frozen cycle [*n* (%)]	72/102 (70.6)	180/270 (66.7)	0.535	60/105 (57.1)	30/75 (40.0)	0.023

HOMA-IR, the homeostasis model assessment of insulin resistance; IVF, in vitro fertilization; ICSI, intracytoplasmic sperm injection; OHSS, ovarian hyper-stimulation syndrome; CPR, clinical pregnancy rate. p < 0.05 was regarded as statistical different.

### 3.4 Metformin pre-treatment for PCOS patients with HOMA-IR ≥ 2.71

For PCOS patients with HOMA-IR ≥ 2.71, we found no difference in IVF fertilization rate, ICSI fertilization rate, high-quality D3 embryo rate, available blastocyst rate, or high-quality blastocyst rate between the metformin group and the control group. The cleaved oocyte rate (97.5% vs. 98.7%, *p* = 0.009), available D3 embryo rate (71.6% vs. 76.1%, *p* = 0.001), and blastocyst rate (43.2% vs. 50.4%, *p* < 0.001) were statistically lower in the metformin group than in the control group (as shown in **Table 3**).

Among 147 fresh ET cycles (99 in the metformin group and 48 in the control group), the clinical pregnancy rate was comparable in the metformin group and the control group (51.5% vs. 56.3, *p* = 0.590). However, among 180 frozen ET cycles (105 in the metformin group and 75 in the control group), the clinical pregnancy rate in the metformin group was significantly higher than in the control group (57.1% vs. 40.0%, *p* = 0.023). After adjusting for PCOM, T, basal FSH, basal LH, and FAI, there was no statistical difference in the clinical pregnancy rate in fresh ET cycles either. The clinical pregnancy rate in frozen ET cycles was still higher in the metformin group than in the control group (*p* = 0.007) (as shown in **Supplementary Table 5**).

## 4 Discussion

PCOS is the most common endocrinopathy with complex reproductive, metabolic, and psychological manifestations (11). It is challenging for PCOS patients to receive IVF/ICSI-ET because of unsatisfactory COS-related parameters, a lower clinical pregnancy rate, and a higher risk of OHSS, miscarriage, and other pregnancy complications compared with healthy women (4). IR reflects metabolic and mitogenic disorders (12), playing crucial roles in the pathological mechanisms of PCOS, and is closely associated with obesity and hyperandrogenism (13, 14). HOMA-IR is a simple and convenient indicator to evaluate the degree of IR (15). Patients with a higher HOMA-IR were reported to have a lower implantation rate (15), a lower clinical pregnancy rate (15, 16), and a higher risk of early miscarriage (17) and late miscarriage (10) than patients with a lower HOMA-IR in IVF/ICSI-ET cycles. Metformin is a synthetically derived biguanide that is widely used in PCOS patients because it improves insulin sensitivity (18, 19), but the application of metformin is full of controversy. A cohort study indicated no positive role of metformin on the success rate of IVF/ICSI-ET for PCOS patients (20). In addition, a randomized double-blind controlled trial (RCT) exploring the efficacy of pre-treatment of metformin for all PCOS patients who received IVF/ICSI-ET treatment showed no difference in implantation rate, multiple pregnancy rate, miscarriage rate, or live birth rate (21). Similarly, another systematic review and meta-analysis showed a similar clinical pregnancy rate for all PCOS patients with and without metformin pre-treatment during IVF/ICSI-ET cycles (22).

In this study, we retrospectively collected information on IVF/ICSI-ET cycles for 969 PCOS patients and investigated the value of HOMA-IR as a judgment criterion for metformin pre-treatment before IVF/ICSI-ET cycles in patients with PCOS. Clinical pregnancy rate was selected as the primary outcome of this study. For all PCOS patients not grouped according to HOMA-IR, we observed a comparable clinical pregnancy rate in both fresh ET and frozen ET cycles between the metformin group and the control group, which were in correlation with previous studies (22). For PCOS patients with HOMA-IR < 2.71, there was no statistical difference in the clinical pregnancy rate between the metformin group and the control group in both fresh and frozen ET cycles. For PCOS patients with HOMA-IR ≥ 2.71, we observed a higher clinical pregnancy rate in the metformin group than in the control group in frozen ET cycles, which was similar to a previous systematic review and meta-analysis that observed the same outcome in PCOS patients with BMI > 26 kg/m^2^ (22). We can initially conclude that metformin pre-treatment increases the clinical pregnancy rate for PCOS patients with HOMA-IR ≥ 2.71 in frozen IVF/ICSI-ET cycles, which was in agreement with our knowledge that metformin can ameliorate IR and decrease the adverse effects of IR on IVF/ICSI-ET outcomes. However, the difference in the clinical pregnancy rate between the two groups was not significant in fresh ET cycles even for PCOS patients with HOMA-IR ≥ 2.71. We try to explain the results and speculate that metformin pre-treatment may affect the endometrial receptivity of PCOS patients during fresh ET cycles; therefore, the effect of metformin pre-treatment on the clinical pregnancy rate was weakened. During frozen ET cycles, artificial menstrual cycle was established; therefore, the effect of metformin pre-treatment on endometrial receptivity was not shown.

COS-related parameters were selected as secondary outcomes of this study. We have to be concerned that metformin pre-treatment seems to have an important influence on the COS-related parameters. All of the included PCOS patients received the GnRH-antagonist protocol treatment. We found that metformin not only decreased the number of oocytes retrieved, the available D3 embryo rate, the cleaved oocyte rate, and the blastocyst formation rate for all PCOS patients not grouped according to HOMA-IR and PCOS patients with HOMA-IR ≥ 2.71, but also decreased the available D3 embryo rate and the available blastocyst rate for all PCOS patients not grouped according to HOMA-IR and PCOS patients with HOMA-IR < 2.71. The above results were in agreement with a previous RCT, which found that metformin pre-treatment decreased the mean number of the retrieved oocytes and the number of fertilized oocytes (21). Interestingly, although the influence of metformin on COS-related parameters was observed in our study and a previous RCT, no adverse effect of metformin on the clinical pregnancy rate was found. The above results indicated that the influence of metformin pre-treatment on COS-related parameters should not be considered as an adverse effect in IVF/ICSI-ET because COS-related parameters are not correlated with IVF/ICSI-ET outcomes entirely.

There were some limitations preventing the generalization of the results. Firstly, this study was a single-center retrospective cohort study, the grade of clinical evidence was limited, and we did not perform a follow-up investigation on the changes in HOMA-IR, BMI, blood lipid, and other indicators in the metformin group after metformin pre-treatment, which could have been helpful for analyzing the correlation between indicator changes and pregnancy outcome. Secondly, because this is a retrospective study, we could not be involved with the patients; thus, the duration and dosage of metformin pre-treatment for included PCOS patients were different, which may affect the outcomes of this study. Thirdly, we selected 2.71 as the cutoff value of HOMA-IR according to previous reports; whether the cutoff value may affect the outcomes of this study was not clear and needs to be explored in the future, but this study indicated that HOMA-IR was a useful indicator for the guidance of metformin pre-treatment before IVF/ICSI-ET for PCOS patients. The above limitations indicated that we must interpret the results of this study with caution, and a well-designed RCT with a large sample size should be performed to obtain a more valuable conclusion.

In conclusion, metformin pre-treatment could be added for PCOS patients with HOMA-IR ≥ 2.71 during frozen IVF/ICSI-ET cycles to improve the clinical pregnancy rate, but for PCOS patients with HOMA-IR < 2.71 or PCOS patients receiving fresh IVF/ICSI-ET treatment, metformin pre-treatment was not proven to be of benefit by the present study. The influence of metformin on COS-related parameters of PCOS patients should be considered in the GnRH-antagonist protocol. The results of this study need to be proven by a well-designed RCT with and large sample size in the future.

## Data availability statement

The raw data supporting the conclusions of this article will be made available by the authors, without undue reservation.

## Ethics statement

The study was approved by the Ethics Committee of West China Second University Hospital.

## Author contributions

ZL contributed to this study’s conception and design. Material preparation, data collection, and analysis were performed by RG and WM. The first draft of the manuscript was written by RG and LQ, and all authors commented on previous versions of the manuscript. All authors contributed to the article and approved the submitted version.

## References

[1] Goodarzi MO Dumesic DA Chazenbalk G Azziz R. Polycystic ovary syndrome: Etiology, pathogenesis and diagnosis. Nat Rev Endocrinol (2011) 7(4):219–31. doi: 10.1038/nrendo.2010.217 21263450

[2] Chang J Azziz R Legro R Dewailly D Franks R Tarlatzis R. Revised 2003 consensus on diagnostic criteria and long-term health risks related to polycystic ovary syndrome. Fertil Steril (2004) 81(1):19–25. doi: 10.1016/j.fertnstert.2003.10.004 14711538

[3] Balen AH Morley LC Misso M Franks S Legro RS Wijeyaratne CN. The management of anovulatory infertility in women with polycystic ovary syndrome: An analysis of the evidence to support the development of global WHO guidance. Hum Reprod Update (2016) 22(6):687–708. doi: 10.1093/humupd/dmw025 27511809

[4] Sha T Wang X Cheng W Yan Y. A meta-analysis of pregnancy-related outcomes and complications in women with polycystic ovary syndrome undergoing IVF. Reprod BioMed Online (2019) 39(2):281–93. doi: 10.1016/j.rbmo.2019.03.203 31255606

[5] Krentz AJ. Insulin resistance. Bmj (1996) 313(7069):1385–9. doi: 10.1136/bmj.313.7069.1385 PMC2352942 8956710

[6] Joham AE Norman RJ Stener-Victorin E Legro RS Franks S Moran LJ. Polycystic ovary syndrome. Lancet Diabetes Endocrinol (2022) 10(9):668–80. doi: 10.1016/S2213-8587(22)00163-2 35934017

[7] Fica S Albu A Constantin M Dobri GA. Insulin resistance and fertility in polycystic ovary syndrome. J Med Life (2008) 1(4):415–22. PMC3018970 20108521

[8] Matli B Schulz A Koeck T Falter T Lotz J Rossmann H. Distribution of HOMA-IR in a population-based cohort and proposal for reference intervals. Clin Chem Lab Med (2021) 59(11):1844–51. doi: 10.1515/cclm-2021-0643 34380182

[9] Tso LO Costello MF Albuquerque LET Andriolo RB Macedo CR. Metformin treatment before and during IVF or ICSI in women with polycystic ovary syndrome. Cochrane Database Syst Rev (2020) 12(12):Cd006105. doi: 10.1002/14651858.CD006105.pub4 33347618 PMC8171384

[10] Yang T Yang Y Zhang Q Liu D Liu N Li Y. Homeostatic model assessment for insulin resistance is associated with late miscarriage in non-dyslipidemic women undergoing fresh IVF/ICSI embryo transfer. Front Endocrinol (Lausanne) (2022) 13:880518. doi: 10.3389/fendo.2022.880518 35784578 PMC9247267

[11] Teede HJ Misso ML Costello MF Dokras A Laven J Moran L. Recommendations from the international evidence-based guideline for the assessment and management of polycystic ovary syndrome. Hum Reprod (2018) 33(9):1602–18. doi: 10.1093/humrep/dey256 PMC6112576 30052961

[12] American Diabetes Association. Standards of medical care in diabetes-2015 abridged for primary care providers. Clin Diabetes (2015) 33(2):97–111. doi: 10.2337/diaclin.33.2.97 25897193 PMC4398006

[13] Stepto NK Cassar S Joham AE Hutchison SK Harrison CL Goldstein RF. Women with polycystic ovary syndrome have intrinsic insulin resistance on euglycaemic-hyperinsulaemic clamp. Hum Reprod (2013) 28(3):777–84. doi: 10.1093/humrep/des463 23315061

[14] Meyer C McGrath BP Teede HJ. Overweight women with polycystic ovary syndrome have evidence of subclinical cardiovascular disease. J Clin Endocrinol Metab (2005) 90(10):5711–6. doi: 10.1210/jc.2005-0011 16046590

[15] Song H Yu Z Li P Wang Y Shi Y. HOMA-IR for predicting clinical pregnancy rate during IVF. Gynecol Endocrinol (2022) 38(1):33–8. doi: 10.1080/09513590.2021.1952976 34263713

[16] Chang EM Han JE Seok HH Lee DR Yoon TK Lee WS. Insulin resistance does not affect early embryo development but lowers implantation rate in *in vitro* maturation-*in vitro* fertilization-embryo transfer cycle. Clin Endocrinol (Oxf) (2013) 79(1):93–9. doi: 10.1111/cen.12099 23176069

[17] Chen Y Guo J Zhang Q Zhang C. Insulin resistance is a risk factor for early miscarriage and macrosomia in patients with polycystic ovary syndrome from the first embryo transfer cycle: A retrospective cohort study. Front Endocrinol (Lausanne) (2022) 13:853473. doi: 10.3389/fendo.2022.853473 35498421 PMC9046670

[18] Mastrototaro L Roden M. Insulin resistance and insulin sensitizing agents. Metabolism (2021) 125:154892. doi: 10.1016/j.metabol.2021.154892 34563556

[19] Morley LC Tang T Yasmin E Norman RJ Balen AH. Insulin-sensitising drugs (metformin, rosiglitazone, pioglitazone, d-chiro-inositol) for women with polycystic ovary syndrome, oligo amenorrhoea and subfertility. Cochrane Database Syst Rev (2017) 11(11):Cd003053. doi: 10.1002/14651858.CD003053.pub6 29183107 PMC6486196

[20] Kalem MN Kalem Z Gurgan T. Effect of metformin and oral contraceptives on polycystic ovary syndrome and IVF cycles. J Endocrinol Invest (2017) 40(7):745–52. doi: 10.1007/s40618-017-0634-x 28244019

[21] Abdalmageed OS Farghaly TA Abdelaleem AA Abdelmagied AE Ali MK Abbas AM. Impact of metformin on IVF outcomes in overweight and obese women with polycystic ovary syndrome: A randomized double-blind controlled trial. Reprod Sci (2019) 26(10):1336–42. doi: 10.1177/1933719118765985 29576001

[22] Wu Y Tu M Huang Y Liu Y Zhang D. Association of metformin with pregnancy outcomes in women with polycystic ovarian syndrome undergoing *In vitro* fertilization: A systematic review and meta-analysis. JAMA Netw Open (2020) 3(8):e2011995. doi: 10.1001/jamanetworkopen.2020.11995 32744629 PMC7399751

