# Supplementary material for: The homeostasis model assessment of insulin resistance is a judgment criterion for metformin pre-treatment before IVF/ICSI and embryo transfer cycles in patients with polycystic ovarian syndrome
Source: Front Endocrinol (Lausanne). 2023 Feb 9;14:1106276. doi: 10.3389/fendo.2023.1106276 (PMC9946957; doi:10.3389/fendo.2023.1106276)
Supplement: Supplementary file 1 [file Table_1.docx]

Supplementary Table 1. Baseline information and laboratory data between metformin group and control group for PCOS participants with HOMA-IR < 2.71

|  | Metformin group (n=171) | Control group (n=435) | p value |
| --- | --- | --- | --- |
| Age (year) | 30±3 | 29±3 | 0.001 |
| BMI (kg/m2) | 22.48±3.25 | 21.73±3.22 | 0.010 |
| Duration of infertility (year) | 4±3 | 3±2 | 0.005 |
| Type of infertility [n(%)] |  |  | 0.143 |
| Primary | 126 (73.7) | 294 (67.6) |  |
| Secondary | 45 (26.3) | 141 (32.4) |  |
| PCOM [n(%)] | 147 (86.0) | 363 (83.4) | 0.445 |
| history of OC treatment [n(%)] | 126 (73.7) | 159 (36.6) | <0.001 |
| AMH (ng/mL) | 10.52±4.95 | 10.98±5.08 | 0.317 |
| Basal E2 (pg/mL) | 42.2±16.8 | 42.9±17.9 | 0.642 |
| Basal P (ng/mL) | 0.48±0.19 | 0.52±0.21 | 0.057 |
| Basal FSH (IU/L) | 6.1±2.0 | 6.7±1.9 | 0.001 |
| Basal LH (IU/L) | 9.0±6.3 | 9.3±6.2 | 0.549 |
| Basal T (mg/dL) | 0.50±0.18 | 0.47±0.77 | 0.607 |
| Basal DHEAS (ug/dL) | 223.6±74.8 | 238.3±121.8 | 0.147 |
| Basal ASD (ng/mL) | 3.58±1.14 | 3.74±1.45 | 0.300 |
| Basal SHBG (nmol/L) | 38.4±25.9 | 60.2±37.6 | 0.247 |
| FAI | 5.7±3.1 | 3.8±3.3 | <0.001 |
| HCY (umol/L) | 9.73±2.63 | 9.08±2.52 | 0.006 |
| HDL-C (mmol/L) | 1.39±0.38 | 1.39±0.33 | 0.966 |
| TG (mmol/L) | 2.27±2.61 | 1.60±1.98 | 0.003 |
| Type of Gn [n(%)] |  |  | <0.001 |
| rFSH | 72 (42.1) | 270 (62.1) |  |
| hMG | 99 (57.9) | 165 (37.9) |  |
| Starting dosage of Gn (IU) | 169.1±46.6 | 170.8±54.1 | 0.718 |
| Total dosage of Gn (IU) | 1871.6±864.2 | 1690.9±650.5 | 0.014 |
| Duration of Gn (day) | 10±2 | 10±2 | 0.001 |
| On the trigger day |  |  |  |
| E2 (pg/mL) | 4975.1±3035.8 | 5357.2±3116.5 | 0.172 |
| P (ng/mL) | 1.04±0.55 | 1.15±0.53 | 0.025 |
| LH (IU/L) | 2.4±2.0 | 2.4±1.8 | 0.886 |
| Single endometrium thickness (mm) | 5.2±1.0 | 5.2±0.9 | 0.971 |
| No. of follicles ≥ 14 mm | 10±4 | 10±3 | 0.504 |
| Fertility methods [n(%)] |  |  | 0.008 |
| IVF | 153 (89.5) | 342 (78.6) |  |
| ICSI | 6 (3.5) | 30 (6.9) |  |
| IVF+ICSI | 12 (7.0) | 63 (14.5) |  |

BMI: body mass index; HOMA-IR: the homeostasis model assessment of insulin resistance; PCOM: polycystic ovarian morphology; OC: oral contraceptives; AMH: anti- -Mullerian hormone; E2: estradiol; P: progesterone; FSH: follicle-stimulating hormone; LH: luteinizing hormone; T: testosterone; DHEAS: dhas dehydroepiandrosterone sulfate; ASD: androstenedione; SHBG: sex hormone binding globulin; FAI: free androgen index; HCY: homocysteine; HDL-C: high-density lipoprotein cholesterol; TG: triglycerides; Gn: gonadotropin; rFSH: recombinant FSH; hMG: human menopausal gonadotropin; IVF: in vitro fertilization; ICSI: intracellular sperm injection. P<0.05 was regarded as statistical different.

Supplementary Table 2. Baseline information and laboratory data between metformin group and control group for PCOS participants with HOMA-IR ≥ 2.71

|  | Metformin group (n=195) | Control group (n=168) | p value |
| --- | --- | --- | --- |
| Age (year) | 29±4 | 30±4 | 0.721 |
| BMI (kg/m2) | 24.57±3.11 | 25.01±2.69 | 0.158 |
| Duration of infertility (year) | 4±2 | 4±2 | 0.880 |
| Type of infertility [n(%)] |  |  | 0.249 |
| Primary | 123 (63.1) | 96 (57.1) |  |
| Secondary | 72 (36.9) | 72 (42.9) |  |
| PCOM [n(%)] | 156 (92.9) | 156 (80.0) | <0.001 |
| history of OC treatment [n(%)] |  |  |  |
| AMH (ng/mL) | 9.18±4.53 | 9.10±4.71 | 0.879 |
| Basal E2 (pg/mL) | 40.8±14.3 | 42.7±14.5 | 0.205 |
| Basal P (ng/mL) | 0.47±0.19 | 0.51±0.29 | 0.138 |
| Basal FSH (IU/L) | 6.2±1.6 | 7.0±1.5 | <0.001 |
| Basal LH (IU/L) | 8.2±5.2 | 9.8±5.1 | 0.004 |
| Basal T (mg/dL) | 0.55±0.31 | 0.43±0.25 | <0.001 |
| Basal DHEAS (ug/dL) | 261.1±142.0 | 232.3±105.0 | 0.070 |
| Basal ASD (ng/mL) | 3.84±1.51 | 3.87±1.23 | 0.844 |
| Basal SHBG (nmol/L) | 26.2±14.6 | 26.2±14.6 | 0.290 |
| FAI | 9.9±8.5 | 7.1±4.1 | 0.002 |
| HCY (umol/L) | 9.45±2.09 | 9.44±2.06 | 0.951 |
| HDL-C (mmol/L) | 1.29±0.35 | 1.13±0.23 | <0.001 |
| TG (mmol/L) | 1.90±1.11 | 1.84±1.15 | 0.659 |
| Type of Gn [n(%)] |  |  | 0.028 |
| rFSH | 96 (49.2) | 102 (60.7) |  |
| hMG | 99 (50.8) | 66 (39.30 |  |
| Starting dosage of Gn (IU) | 185.8±56.7 | 199.1±54.4 | 0.024 |
| Total dosage of Gn (IU) | 1987.3±745.1 | 2107.8±773.2 | 0.132 |
| Duration of Gn (day) | 10±2 | 10±2 | 0.432 |
| On the trigger day |  |  |  |
| E2 (pg/mL) | 4024.7±2259.0 | 4671.8±2523.5 | 0.010 |
| P (ng/mL) | 1.11±1.15 | 1.16±0.60 | 0.649 |
| LH (IU/L) | 2.9±2.1 | 3.4±2.5 | 0.056 |
| Single endometrium thickness (mm) | 5.1±1.0 | 5.5±1.5 | 0.003 |
| No. of follicles ≥ 14 mm | 10±4 | 10±3 | 0.156 |
| Fertility methods [n(%)] |  |  | 0.095 |
| IVF | 174 (89.2) | 144 (85.7) |  |
| ICSI | 3 (1.5) | 0 (0.0) |  |
| IVF+ICSI | 18 (9.2) | 24 (14.3) |  |

BMI: body mass index; HOMA-IR: the homeostasis model assessment of insulin resistance; PCOM: polycystic ovarian morphology; OC: oral contraceptives; AMH: anti- -Mullerian hormone; E2: estradiol; P: progesterone; FSH: follicle-stimulating hormone; LH: luteinizing hormone; T: testosterone; DHEAS: dhas dehydroepiandrosterone sulfate; ASD: androstenedione; SHBG: sex hormone binding globulin; FAI: free androgen index; HCY: homocysteine; HDL-C: high-density lipoprotein cholesterol; TG: triglycerides; Gn: gonadotropin; rFSH: recombinant FSH; hMG: human menopausal gonadotropin; IVF: in vitro fertilization; ICSI: intracellular sperm injection. P<0.05 was regarded as statistical different.

Supplementary Table 3. Adjusted clinical pregnancy rate for all PCOS participants

|  | Adjusted OR (95%CI) | Adjusted p value |
| --- | --- | --- |
| Fresh CPR | 0.873 (0.367-2.076) | 0.759 |
| Frozen CPR | 0.790 (0.409-1.528) | 0.484 |

Adjusting for age, body mass index, duration of infertility, history of oral contraceptives treatment, anti-Mullerian hormone, basal follicle-stimulating hormone, basal luteinizing hormone, free androgen index, homocysteine and triglyceride. Control group= “0”, metformin group= “1”. P<0.05 was regarded as statistical different.

Supplementary Table 4. Adjusted clinical pregnancy rate for PCOS participants with HOMA-IR < 2.71

|  | Adjusted OR (95%CI) | Adjusted p value |
| --- | --- | --- |
| Fresh CPR | 1.499 (0.250-8.875) | 0.658 |
| Frozen CPR | 0.703 (0.327-1.510) | 0.366 |

Adjusting for age, body mass index, duration of infertility, history of oral contraceptives treatment, basal follicle-stimulating hormone, free androgen index, homocysteine and triglyceride. Control group= “0”, metformin group= “1”. P<0.05 was regarded as statistical different.

Supplementary Table 5. Adjusted clinical pregnancy rate for PCOS participants with HOMA-IR ≥ 2.71

|  | Adjusted OR (95%CI) | Adjusted p value |
| --- | --- | --- |
| Fresh CPR | 1.170 (0.418-3.273) | 0.765 |
| Frozen CPR | 4.932 (1.271-19.140) | 0.021 |

Adjusting for polycystic ovarian morphology, testosterone, basal follicle-stimulating hormone, basal luteinizing hormone, free androgen index. Control group= “0”, metformin group= “1”. P<0.05 was regarded as statistical different.
